# Comparative effect of iso-osmolar versus low-osmolar contrast media on the incidence of contrast-induced acute kidney injury in diabetic patients: a systematic review and meta-analysis

**DOI:** 10.1186/s40644-019-0224-6

**Published:** 2019-06-18

**Authors:** Fei Zhao, Rong Lei, Shi-Kun Yang, Min Luo, Wei Cheng, Ye-Qing Xiao, Xu-Wei Li, Jun Guo, Shao-Bin Duan

**Affiliations:** 1 Department of Nephrology, The Second Xiangya Hospital, Central South University, 139 Renmin Road, Changsha, 410011 Hunan People’s Republic of China; 2 Department of Nephrology, Changsha Central hospital, Changsha, 410004 Hunan People’s Republic of China; 3 Department of Nephrology, The Third Xiangya Hospital, Central South University, Changsha, 410013 Hunan People’s Republic of China

**Keywords:** Acute kidney injury, Diabetes, Contrast media

## Abstract

**Background:**

Contrast-induced acute kidney injury (CI-AKI) is a major adverse effect caused by intravascular administration of iodinated contrast medium. Whether there is a difference in CI-AKI incidence between iso-osmolar (IOCM) and low-osmolar contrast media (LOCM) among diabetic patients is controversial.

**Methods:**

Randomized controlled trials comparing the nephrotoxic effects between IOCM and LOCM in diabetic patients with or without CKD (eGFR< 60 ml/min/1.73 m^2^) were included in the analysis. The incidence of CI-AKI was defined as an initial increase in serum creatinine (SCr) concentration of at least 0.5 mg/dl or a rise in creatinine of 25% from baseline.

**Results:**

A total of 2190 patients were included, among whom 1122 patients received IOCM and 1068 received LOCM. When compared to LOCM, IOCM had no significant benefit in preventing CI-AKI (OR = 1.66, [CI: 0.97–2.84], *P* = 0.06, I^2^ = 54%). However, the difference between IOCM and LOCM was found when CI-AKI was defined as an absolute SCr increase (≥0.5 mg/dl) rather than a relative SCr increase (≥25%). Further analysis showed that LOCM resulted in more adverse events.

**Conclusions:**

Whether there is a difference of CI-AKI incidence between IOCM and LOCM in diabetic patients was related to the selected diagnostic criteria. The incidence of adverse events was significantly lower with IOCM when compared with LOCM. Therefore, we suggest that IOCM may be used in diabetic and CKD (eGFR< 60 ml/min/1.73 m^2^) patients.

**Electronic supplementary material:**

The online version of this article (10.1186/s40644-019-0224-6) contains supplementary material, which is available to authorized users.

## Background

Contrast-induced acute kidney injury (CI-AKI) is a severe complication of exposure to iodine contrast media for diagnostic or interventional procedure [1], which accounts for increase in morbidity, mortality, length of stay and hospitalization cost [2]. Controversy remains whether certain contrast media types with various osmolarities are associated with a lower risk of CI-AKI [3, 4]. Although much progress has been made to improve the quality of contrast media, acute kidney injury after intravascular contrast administration remains a major concern for clinicians.

The literatures contain conflicting reports about whether IOCM is associated with less risk for CI-AKI than LOCM [3, 5]. According to the international guidelines from both European Society of Urogenital Radiology (ESUR) and the Kidney Disease: Improving Global Outcomes guidelines (KIDGO), Both IOCM and LOCM were recommended in patients with increased risk of CI-AKI [6, 7]. Diabetic (DM) is one of the most important public health challenges in the twenty-first century. [8]. The risk for CI-AKI is significantly increased in patients with chronic kidney disease (CKD), especially when DM coexists [9]. Several studies have focused on the nephrotoxic comparison between IOCM and LOCM in diabetic patients [3, 10, 11]. However, there are still uncertainties on whether there are any significant differences in renal safety between IOCM and LOCM.

We performed a systematic review of randomized, controlled trials (RCTs) to compare the effects of IOCM and LOCM on CI-AKI incidence and the adverse effects in diabetic patients with or without CKD. We hypothesized that with more recent RCTs included in our updating reviews, we could better understand the conflicting results on CI-AKI risk. To our knowledge, this is the first meta-analysis reporting difference in CI-AKI between IOCM and LOCM among DM patients with or without CKD (eGFR < 60 ml/min/1.73 m^2^).

## Methods

### Data collection and search strategy

A literature review on published RCTs was performed using PubMed, the Cochrane Library, and Web of Science until June 2017. Search keywords included “iso-osmolar”, “iodixanol”, “visipaque”, “IOCM” and “diabetes” as MeSH and free text terms. In addition, root variations of the mentioned keywords were used in order to improve search outcomes. Related articles were used as well to broaden the search, and the computer search was supplemented with manual searches of the reference lists of all retrieved studies, review articles, and conference abstracts. The systematic review was conducted according to the Preferred Reporting Items in Systematic Reviews and Meta-analysis (PRISMA) guidelines [12].

### Study selection

Selected studies included prospective randomized controlled comparisons of CI-AKI incidence between IOCM and LOCM in diabetic patients with or without CKD. We only included full-text articles with at least two arms of parallel comparisons. Non-randomized controlled studies, studies exploring CI-AKI incidence during procedures other than diabetes, editorials, letters to the editor, reviews, animal experimental studies and those only published as conference abstracts were excluded. Two reviewers independently screened titles and abstracts to identify articles for inclusion. If necessary, the full texts of articles were reviewed. Discrepancies remained after reviewing the full-text were resolved by consensus. At random intervals during screening, quality checks were performed to ensure that inclusion criteria were applied in consistence. All researches were limited to studies in humans.

### Outcomes

The primary outcome was the incidence of CI-AKI in subjects receiving IOCM versus LOCM. CI-AKI is defined by an initial increase in SCr concentration of at least 0.5 mg/dl or by a relative increase of at least 25% from baseline within 36–72 h after exposure. The numbers of adverse events in the two aforementioned groups were the secondary outcome of this study.

### Data extraction and quality assessment

For every included study, information including study characteristics, study population, imaging procedure type, comparisons, results, and statistical analysis were obtained by one researcher. The extracted information was confirmed for accuracy by another researcher. Discrepancies between the two researchers were resolved by consensus. Trial bias risk and quality assessment of all RCTs were conducted according to the Cochrane collaboration criteria, which pay emphasis on evaluating adequacy of sequence generation, allocation sequence concealment, blinding of participants and caregivers, blinding for outcome assessment, incomplete outcome, selective outcome reporting, and other potential bias. Discrepancies were resolved by consensus.

### Statistical analysis

All analyses were performed using Review Manager (RevMan) Version 5.3 for Windows (Oxford, England). Preferred Reporting Items for Systematic Reviews and Meta-analysis Protocols (PRISMA-P) criteria were used for analysis [13]. Continuous and dichotomous variables were compared by the weighted mean difference (WMD) and odds ratio (OR) respectively.

Chi-square and the I^2^ statistic that describe the percentage of total variation across studies were used to assess heterogeneity. I^2^ value ranges from 0% (no heterogeneity) to 100% (maximal heterogeneity). Chi-square *p*-values of < 0.1 and I^2^ > 50% or 0.5 mean significant heterogeneity. DerSimonian and Laird’s random-effect model was applied for analysis when heterogeneity among studied were high. A *P* value ≤0.05 was considered significant. Publication bias and skewness were evaluated graphically using a funnel plot. Subgroups analyses for comparisons between iodixanol and specific types of LOCM (eg, iohexol) or between specific groups of patients (i.e., those receiving arterial or intravenous injection, use of NAC, volume of contrast media, with or without CKD and those with or without coronary angiography) were outlined before data collection. Post hoc subgroup analyses of studies stratified by the definition of CI-AKI (i.e., > 0.5 mg/dL or > 25% increase from baseline creatine value) were performed when data were available.

## Results

### Identification of studies

Two hundred twenty two potential studies were included in initial search, among which 186 were eliminated based on the titles and abstracts. Remaining articles were identified by scanning through abstracts and excluded based on whether or not including CI-AKI incidence rates in diabetic patients. One article was identified through other references. Fifteen RCTs that compared IOCM to LOCM fulfilled our inclusion criteria (Additional file 1: Figure S1).

### Characteristics of selected clinical trials

All 15 trials used iodixanol as IOCM, iopromide, iopamidol, iohexol, ioversol, and ioxaglate were used as LOCM. Baseline characteristics of the included15 RCTs were presented in Table 1. All data were acquired from diabetic patients with or without CKD. The definition of CI-AKI differed among the trials. CI-AKI was defined as an absolute increase of baseline creatinine by at least 0.5 mg/dl or as a relative increase by at least 25% in most studies. A few studies used both definitions.

**Table 1:**
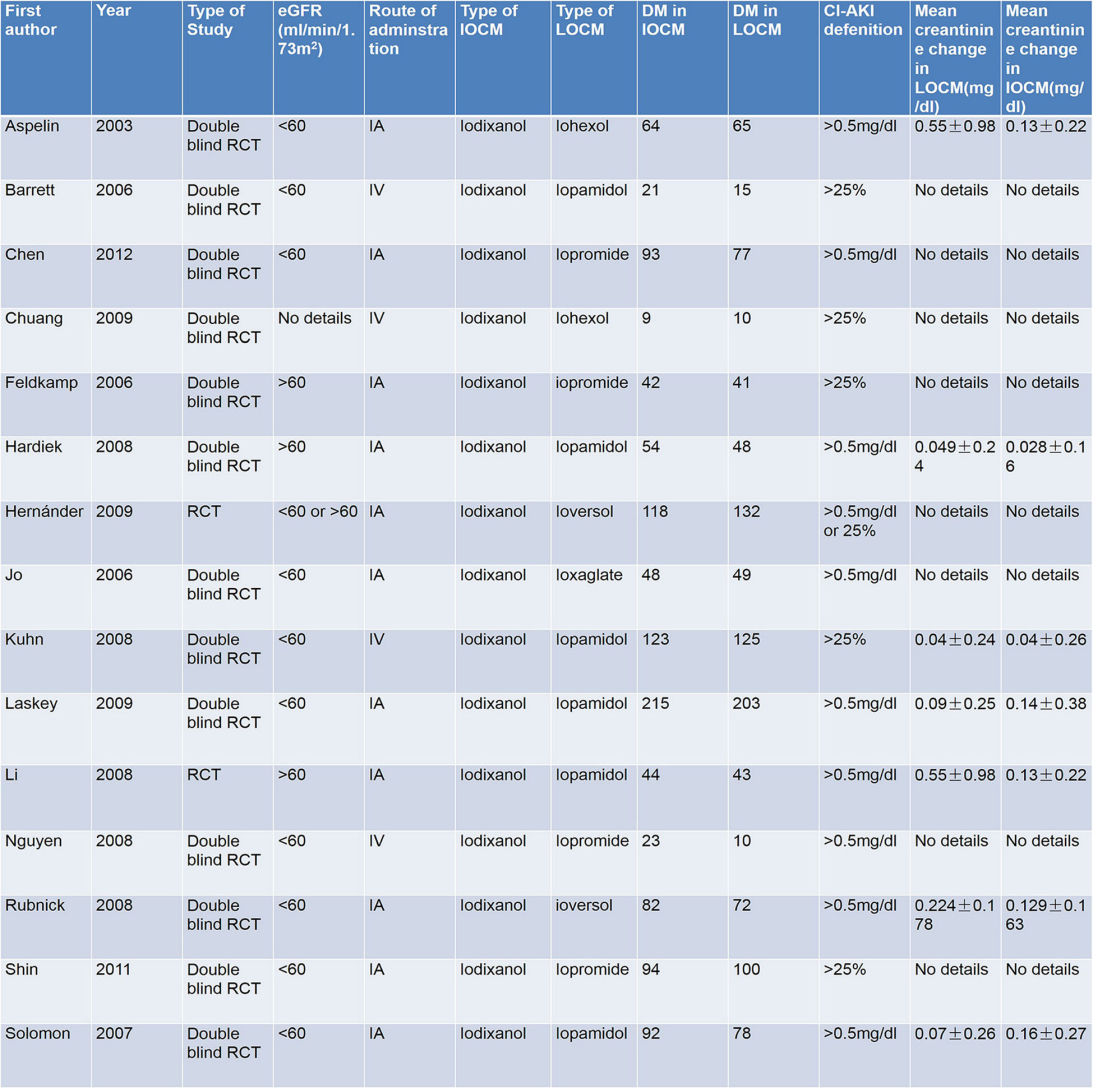
Baseline characteristics of all trials

### Primary outcome

**Fig. 1:**
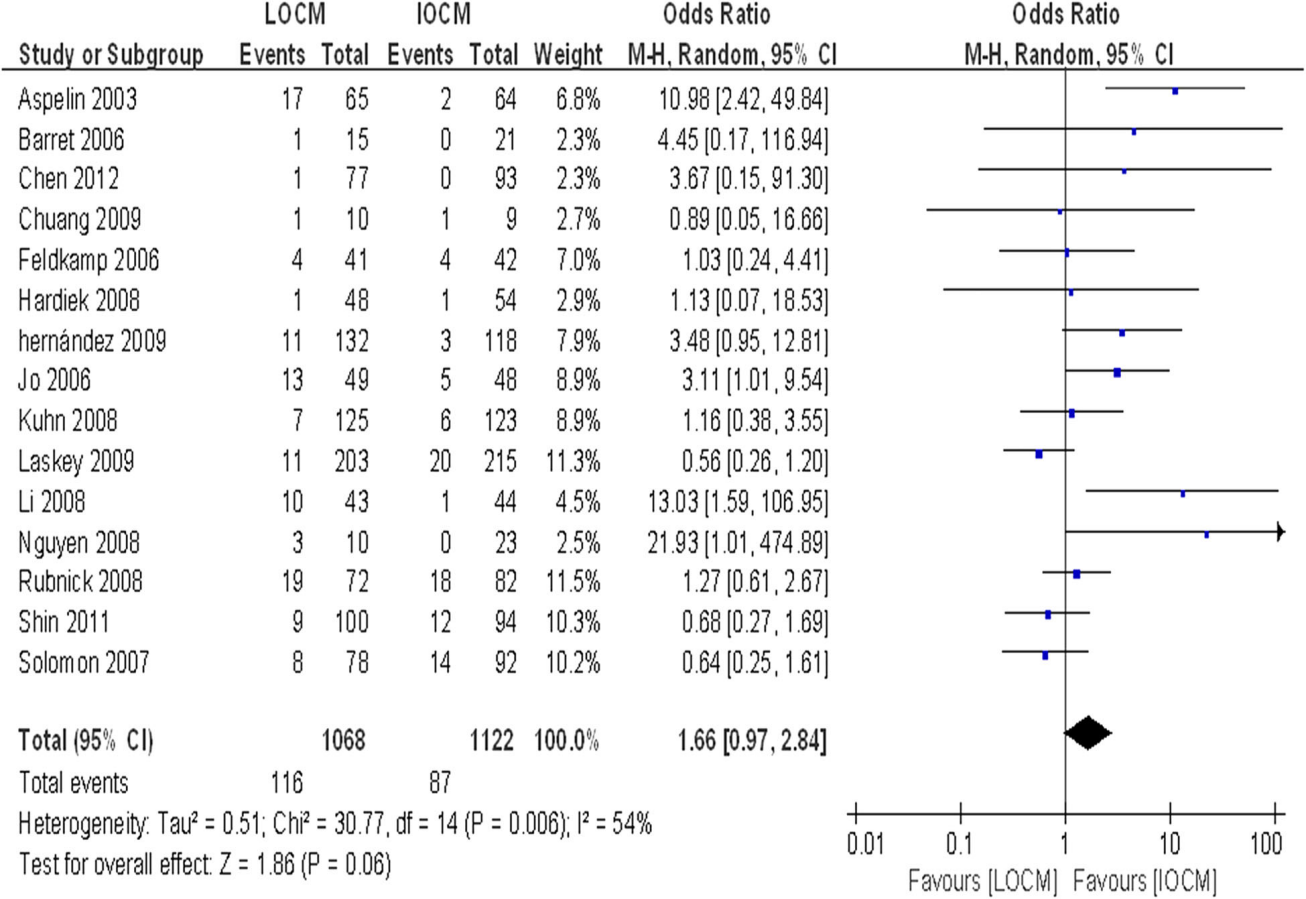
Comparison of the outcomeof CI-AKI between IOCM and LOCM. Odds ratio for individual studies (squares) and meta-analysis (diamonds) and 95% CI (horizontal lines) are shown

**Fig. 2:**
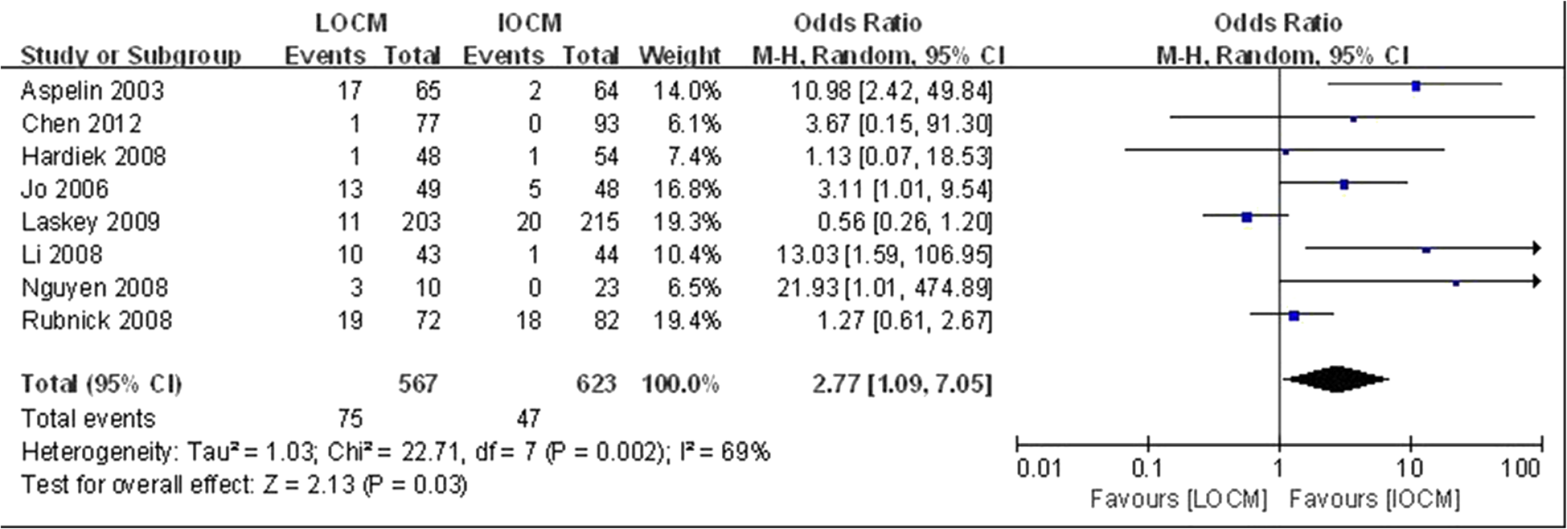
IOCM vs. LOCM for the outcome of CI-AKI (defined by an initial increase in SCr concentration of at least 0.5 mg/dl within 36–72 h of exposure). Odds ratio for individual studies (squares) and meta-analysis (diamonds) and 95% CI (horizontal lines) are shown

To evaluate CI-AKI incidence of IOCM and LOCM in diabetic patients. A total of 2190 patients were included in 15 trials [3, 10, 11, 14–25], among whom 1122 patients used IOCM and 1068 received LOCM. When compared to LOCM, IOCM was not associated with a significantly lower incidence of CI-AKI (OR = 1.66, CI: 0.97–2.84, *P* = 0.06, I^2^ = 54%) (Fig. 1). There was no significant difference of peak increase in one-week serum creatinine between LOCM and IOCM (*P* > 0.05) (Additional file 2: Figure S2). However, the difference between IOCM and LOCM was found when CI-AKI was defined as an absolute SCr increase (≥0.5 mg/dl) (Fig. 2), rather than a relative SCr increase (≥25%) (Additional file 3: Figure S3).

We further performed subgroup analysis based on use of different type of LOCM, contrast volume, N-acetylcysteine (NAC), eGFR, route of administration, and the examination of coronary angiography. The subgroups defined before data collection were based on uniformly reported information. No definitive evidence of a difference in CI-AKI incidence between different types of LOCM was observed (Additional file 4: Figure S4). There was also no significant difference of CI-AKI incidence in terms of volume of contrast media (Additional file 5: Figure S5). There were emerging data showing that use of NAC had no protective effect in the preprocedural preparations, our study showed similar results (Additional file 6: Figure S6) in diabetic patients. We also found that there was no difference in CI-AKI incidence among studies including all CKD patients versus those individuals without CKD (Additional file 7: Figure S7). Furthermore, subgroup analysis based on route of administration (Additional file 8: Figure S8) and the examination of coronary angiography (Additional file 9: Figure S9) did not show any statistically significant difference.

### Secondary outcome

**Fig. 3:**
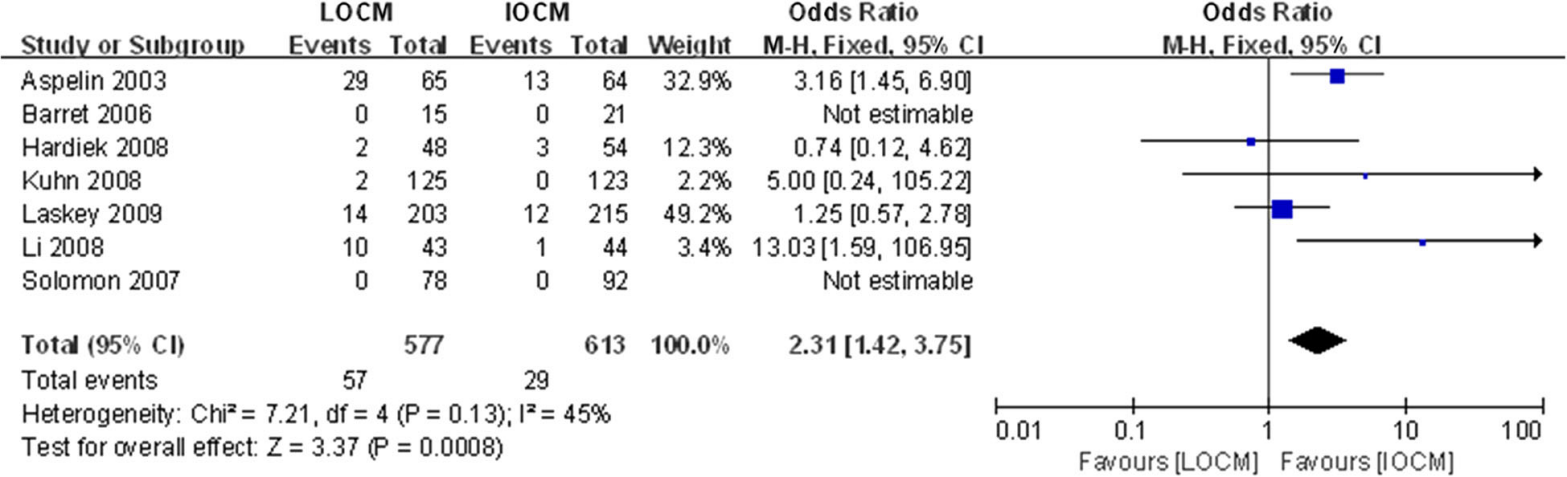
IOCM vs. LOCM for the outcome of adverse events. Odds ratio for individual studies (squares) and meta-analysis (diamonds) and 95% CI (horizontal lines) are shown

To evaluate adverse events induced by IOCM and LOCM in diabetic patients, we selected 7 studies including adverse events. The results showed that there was lower risk of adverse events in IOCM, compared with LOCM in diabetic patients (Fig. 3).

### Investigation of heterogeneity and publication bias

We performed subgroup analysis to explore the effect of IOCM and LOCM on CI-AKI incidence, the analysis was stratified according to patients’ characters and other factors. In brief, there was no significant difference of CI-AKI incidence between IOCM and LOCM. Funnel plots for some key outcomes (Additional file 12: Table S1) suggested there was publication bias among these studies (Additional files 10 and 11: Figures S10-S11.

## Discussion

This meta-analysis of 15 RCTs including 2190 patients comparing the effect between IOCM and LOCM on the incidence of CI-AKI in diabetic patients shown that the use of IOCM has no significant benefit over LOCM in preventing CI-AKI in diabetic patients with or without CKD when CI-AKI was defined as an absolute SCr increase (≥0.5 mg/dl) or a relative SCr increase (≥25%). However, when CI-AKI was defined as an absolute increase of SCr (≥0.5 mg/dl), the CI-AKI incidence of IOCM was lower than that of LOCM. More importantly, IOCM was associated with lower risk of adverse events, compared with LOCM.

CI-AKI is a major adverse effect caused by intravascular administration of iodinated contrast media. In the NEPHRIC trial, more than 130 patients who had CKD and DM performing angiography were prospectively randomized to receive either iodixanol or iohexol, iodixanol is a safer agent, at least in those at higher risk of CI-AKI (defined by an initial increase in SCr level ≥ 0.5 mg/dl), such as those with chronic renal failure due to diabetes mellitus [3]. In contrast, other studies demonstrated that there existed no significant difference in CI-AKI incidence (defined by an initial increase in SCr level ≥ 0.5 mg/dl) between IOCM and LOCM in high risk patients [11]. Studies comparing nephrotoxicity of IOCM and LOCM reported a controversial conclusion [26–28]. A prior meta-analysis suggested that IOCM had no significant difference in the incidences of post-procedure hemodialysis or death over LOCM [29]. Our meta-analysis found no statistical significance of CI-AKI risk with iodixanol compared with LOCM in diabetic patients based on two diagnostic criteria. But IOCM seems safer when referring to an absolute SCr increase and adverse events which indicated that LOCM indeed had an increased nephrotoxic potential compared to iodixanol. Therefore, our results indicated IOCM would be safer in diabetic and CKD patients. Whether there is a significant difference of LOCM and IOCM induced AKI is related with the use of different diagnostic criteria.

We further did subgroup analysis. In the meta-analysis, we did not see any definitive evidence showing difference in CI-AKI incidence between IOCM and LOCM regardless of patient characteristics (with or without CKD) or contrast media volume. Our study confirmed the result from Kooiman et al. that there seemed to be no association between volume of contrast media and CI-AKI [30]. As other studies, use of IOCM showed non-significant benefit in preventing CI-AKI when compared to LOCM in high risk patients [21, 28]. We also found no difference in CI-AKI risk with iodixanol compared with a diverse group of LOCM. However, Reed et al. found iodixanol had a lower CI-AKI incidence when compared with iohexol or ioxaglate, similar result was not obtained between the comparison of IOCM with iopromide, iopamidol, iomeprol, or ioversol [31]. The possible reasons may be that different types of patients were included. Subgroup analysis based on administration route showed the pooled relative risk for the intra-arterial route was 1.57 (CI, 0.82–3.03; *P* = 0.17) and 1.99 (CI, 0.58–6.87; *P* = 0.27) for the intravenous route, suggesting no difference in CI-AKI risk between route of administration. This result was consistent with Eng J’s findings [32]. NAC has long been used as a method to prevent CI-AKI. However, our results indicated that use of NAC showed no protective effect of CI-AKI in diabetic patients, which were similar with other studies [33, 34]. A recent study showed ionic LOCM ioxaglate was associated with a numerically lower one-year mortality than iodixanol in patients with cardiac catheterization [35], which were inconsistent with our results. Therefore, future studies evaluating long-term safety following exposure to different types of contrast media in diabetic patients are warranted.

### Strength and limitations

Up to date, this is the first systematic review and meta-analysis making comparison between IOCM and LOCM on renal safety in diabetic patients. All included studies are RCTs, accounting for very low risk of selection bias. Despite the comprehensiveness and the robust statistical methods in our study, we still have some limitations. First, results of our study are based on the combined data of many heterogeneous randomized, controlled trials. Second, varied definitions of CI-AKI were used in some studies, however, we used only standard definitions according to KDIGO or ESUR guidelines. Third, data could not be fully extracted because of missing information as in any meta-analysis. We tried to contact the studies’ corresponding authors but unfortunately failed. At last, there were evidences of publication bias in some of the subgroup analysis, and therefore the results should be interpreted with caution. The number of included RCTs was limited by the presence of literatures comparing effects of IOCM and LOCM on DM and above inclusion.

## Conclusions

In conclusion, whether there is a difference of CI-AKI incidence between IOCM and LOCM in diabetic patients was related to the selected diagnostic criteria. And the incidence of total adverse events was significantly lower with IOCM when compared with LOCM, such as ischemic stroke events, cardiovascular events, rash, burn sensation in the throat, nausea, vomiting, edema, oliguria, progression to end-stage renal disease and so on. We suggest that IOCM may be used in diabetic and CKD (eGFR< 60 ml/min/1.73 m^2^) patients. This study will provide a scientific guide for clinicians to choose the type of contrast agent in diabetic and CKD patients. However, multi-centers prospective randomized controlled trials are still necessary to evaluate effect of IOCM and LOCM on CI-AKI incidence and long-term outcome in diabetic and CKD patients.

## Additional files

Additional file 1: **Figure S1.** Flow chart of evidence research and selection. (TIF 2140 kb)

Additional file 2: **Figure S2.** IOCM vs. LOCM for the outcome of one-week peak SCr increase. Mean difference for individual studies (squares) and meta-analysis (diamonds) and 95% CI (horizontal lines) are shown. (TIF 2613 kb)

Additional file 3: **Figure S3.** IOCM vs. LOCM for the outcome of CI-AKI (defined by a relative increase of at least 25% from baseline within 36–72 h of exposure). Odds ratio for individual studies (squares) and meta-analysis (diamonds) and 95% CI (horizontal lines) are shown. (TIF 1747 kb)

Additional file 4: **Figure S4.** IOCM vs. LOCM for the outcome of CI-AKI: subgroup analysis based on different types of LOCM. Odds ratio for individual studies (squares) and meta-analysis (diamonds) and 95% CI (horizontal lines) are shown. (TIF 11257 kb)

Additional file 5: **Figure S5.** IOCM vs. LOCM for the outcome of CI-AKI: subgroup analysis based on contrast of volume. Mean difference for individual studies (squares) and meta-analysis (diamonds) and 95% CI (horizontal lines) are shown. (TIF 2726 kb)

Additional file 6: **Figure S6.** IOCM vs. LOCM for the outcome of CI-AKI: subgroup analysis based on using of NAC. Odds ratio for individual studies (squares) and meta-analysis (diamonds) and 95% CI (horizontal lines) are shown. (TIF 3317 kb)

Additional file 7: **Figure S7.** IOCM vs. LOCM for the outcome of CI-AKI: subgroup analysis based on eGFR. Odds ratio for individual studies (squares) and meta-analysis (diamonds) and 95% CI (horizontal lines) are shown. (TIF 3727 kb)

Additional file 8: **Figure S8.** IOCM vs. LOCM for the outcome of CI-AKI: subgroup analysis based on route of administration. Odds ratio for individual studies (squares) and meta-analysis (diamonds) and 95% CI (horizontal lines) are shown. (TIF 3626 kb)

Additional file 9: **Figure S9.** IOCM vs. LOCM for the outcome of CI-AKI: subgroup analysis based on whether or not CAG was performed. Odds ratio for individual studies (squares) and meta-analysis (diamonds) and 95% CI (horizontal lines) are shown. (TIF 3397 kb)

Additional file 10: **Figure S10.** Risk of bias summary: review authors' judgements about each risk of bias item for each included study (TIF 4669 kb)

Additional file 11: **Figure S11.** Risk of bias graph: review authors' judgements about each risk of bias item presented as percentages across all included studies. (TIF 8393 kb)

Additional file 12: **Table S1.** Effect of IOCM and LOCM on CI-AKI incidence among diabetic patients. (TIF 2221 kb)

## Data Availability

The datasets used and/or analyzed during the current study are available from the corresponding author on reasonable request.

## Abbreviations

CAG: Coronary angiography
CI: Confidence interval
CI-AKI: Contrast-induced acute kidney injury
CKD: Chronic kidney disease
DM: Diabetes mellitus
eGFR: Estimated glomerular rate
ESUR: European Society of Urogenital Radiology
IA: Intraarterial administration
IOCM: Iso-osmolar contrast media
IV: Intravenous administration
KIDGO: The Kidney Disease: Improving Global Outcomes
LOCM: Low-osmolar contrast media
NAC: N- acetylcysteine
OR: Odds ratio
PRISMA-P: Systematic reviews and meta-analysis protocols criteria
RCTs: Randomized controlled trials
RevMan: Review Manager
SCr: Serum creatinine
WMD: Weighted mean difference
