# Supplementary figures and images for: Comparative effect of iso-osmolar versus low-osmolar contrast media on the incidence of contrast-induced acute kidney injury in diabetic patients: a systematic review and meta-analysis
Source: Cancer Imaging. 2019 Jun 18;19:38. doi: 10.1186/s40644-019-0224-6 (PMC6580528; doi:10.1186/s40644-019-0224-6)

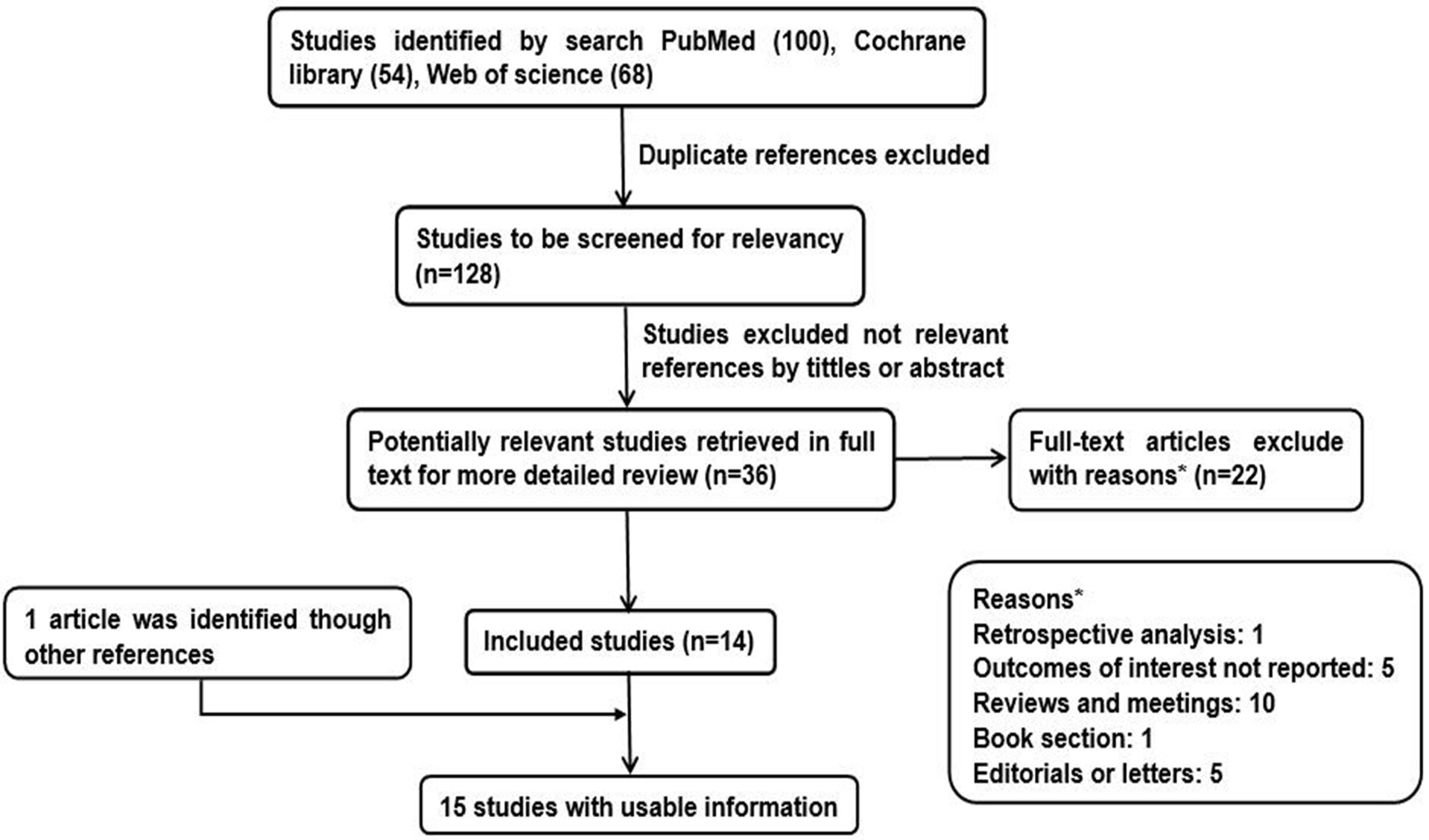

Supplement: Supplementary file 1 — Figure S1. Flow chart of evidence research and selection. (TIF 2140 kb) [file 40644_2019_224_MOESM1_ESM.tif]

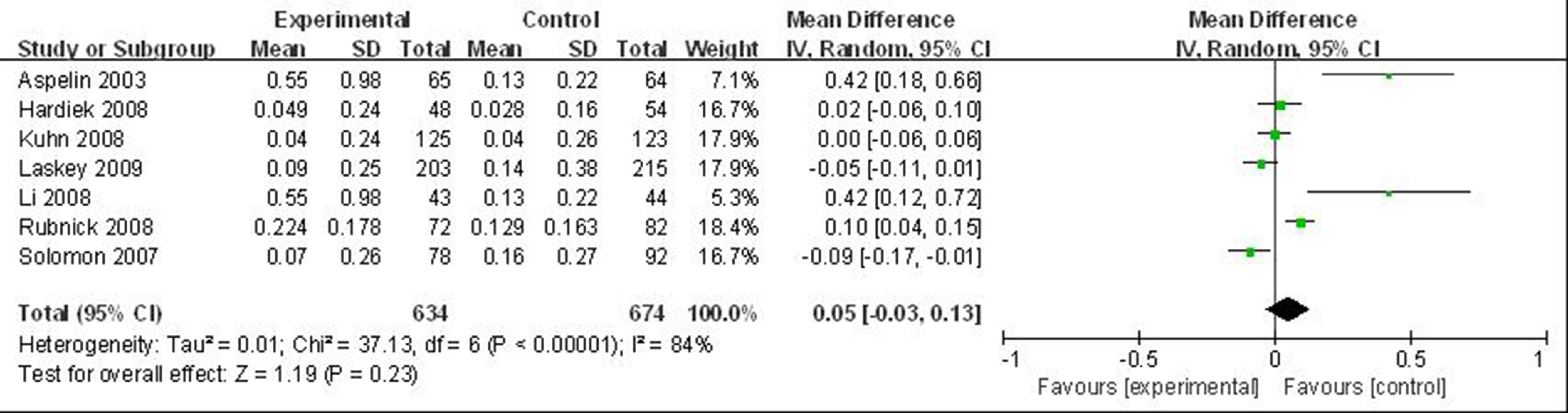

Supplement: Supplementary file 2 — Figure S2. IOCM vs. LOCM for the outcome of one-week peak SCr increase. Mean difference for individual studies (squares) and meta-analysis (diamonds) and 95% CI (horizontal lines) are shown. (TIF 2613 kb) [file 40644_2019_224_MOESM2_ESM.tif]

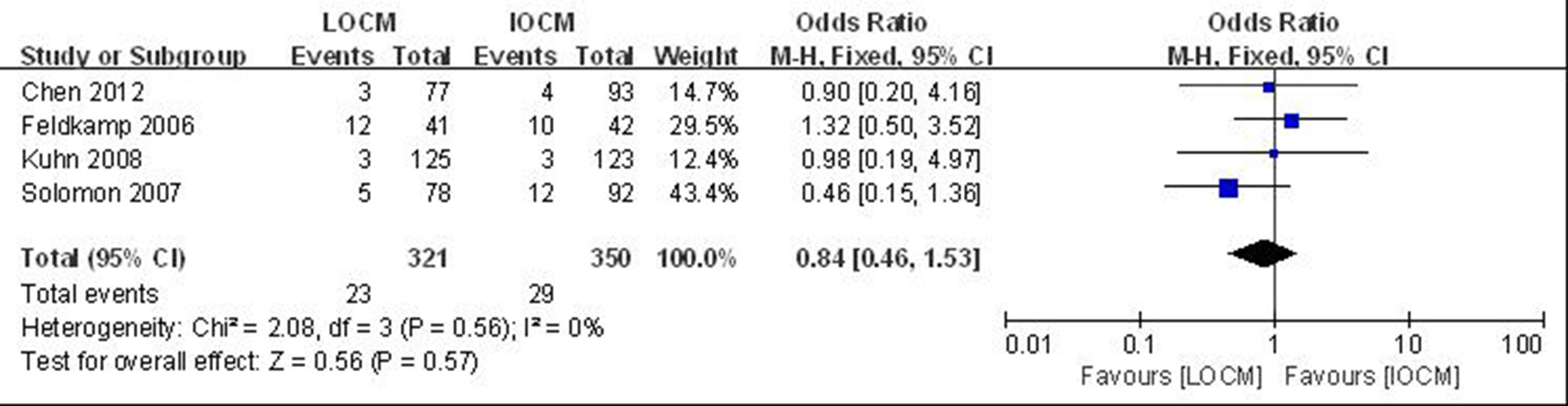

Supplement: Supplementary file 3 — Figure S3. IOCM vs. LOCM for the outcome of CI-AKI (defined by a relative increase of at least 25% from baseline within 36–72 h of exposure). Odds ratio for individual studies (squares) and meta-analysis (diamonds) and 95% CI (horizontal lines) are shown. (TIF 1747 kb) [file 40644_2019_224_MOESM3_ESM.tif]

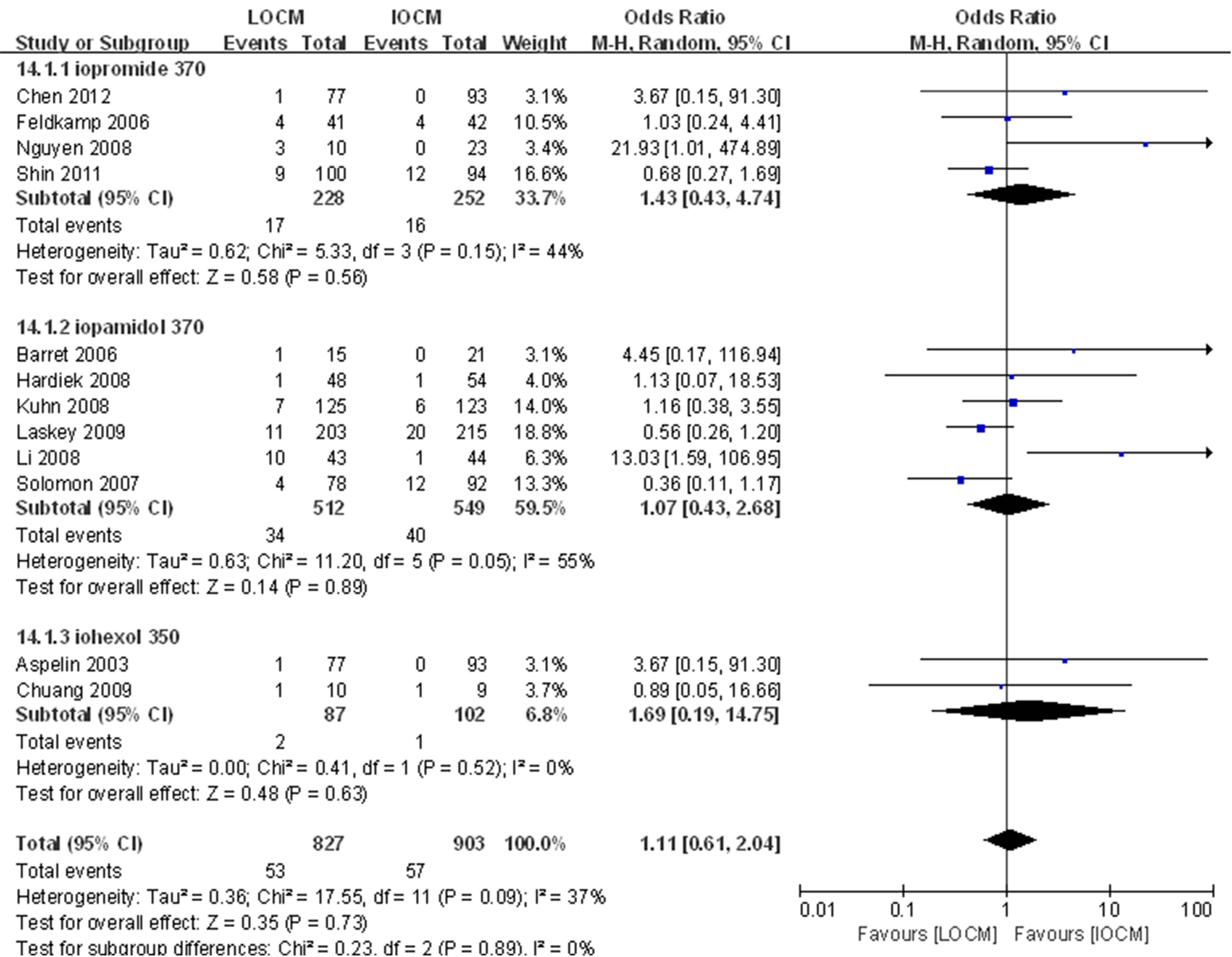

Supplement: Supplementary file 4 — Figure S4. IOCM vs. LOCM for the outcome of CI-AKI: subgroup analysis based on different types of LOCM. Odds ratio for individual studies (squares) and meta-analysis (diamonds) and 95% CI (horizontal lines) are shown. (TIF 11257 kb) [file 40644_2019_224_MOESM4_ESM.tif]

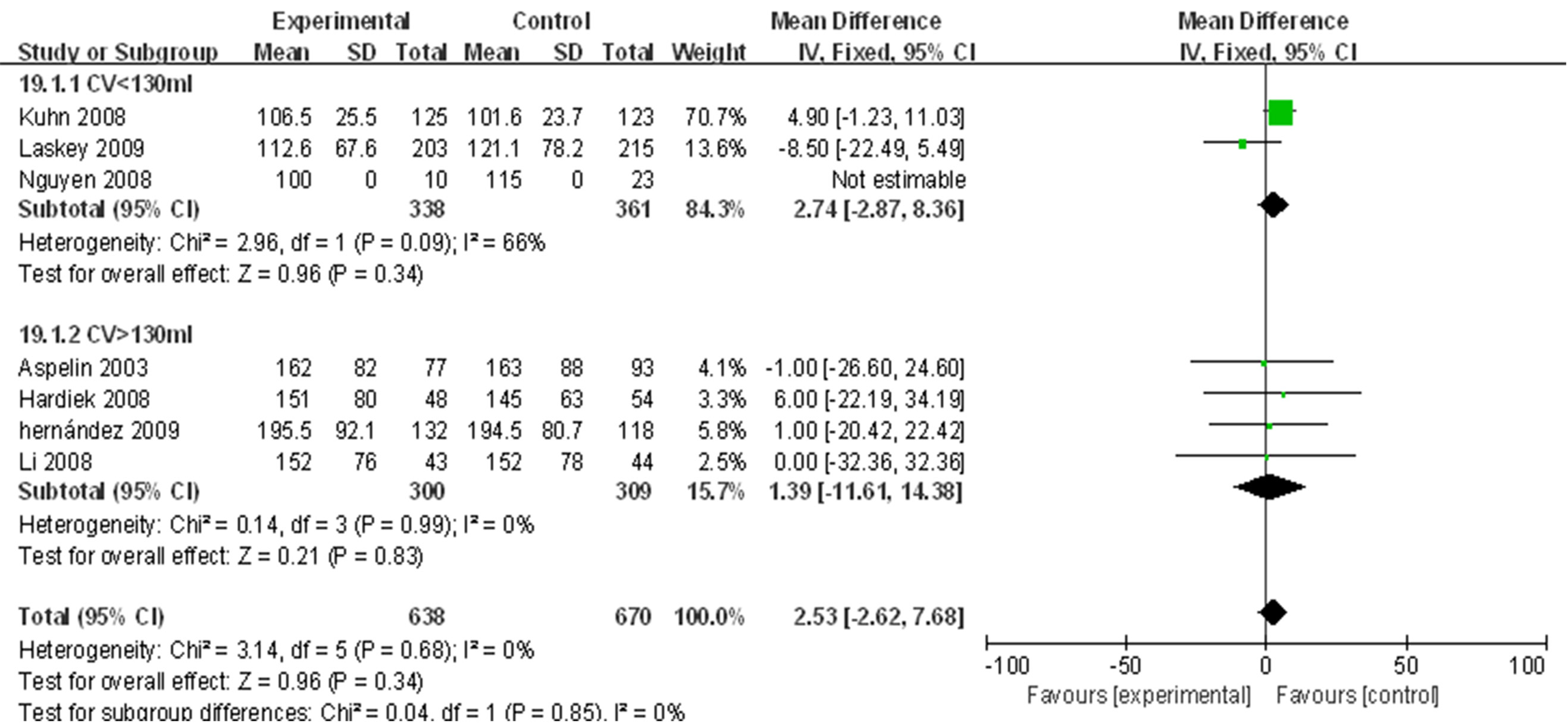

Supplement: Supplementary file 5 — Figure S5. IOCM vs. LOCM for the outcome of CI-AKI: subgroup analysis based on contrast of volume. Mean difference for individual studies (squares) and meta-analysis (diamonds) and 95% CI (horizontal lines) are shown. (TIF 2726 kb) [file 40644_2019_224_MOESM5_ESM.tif]

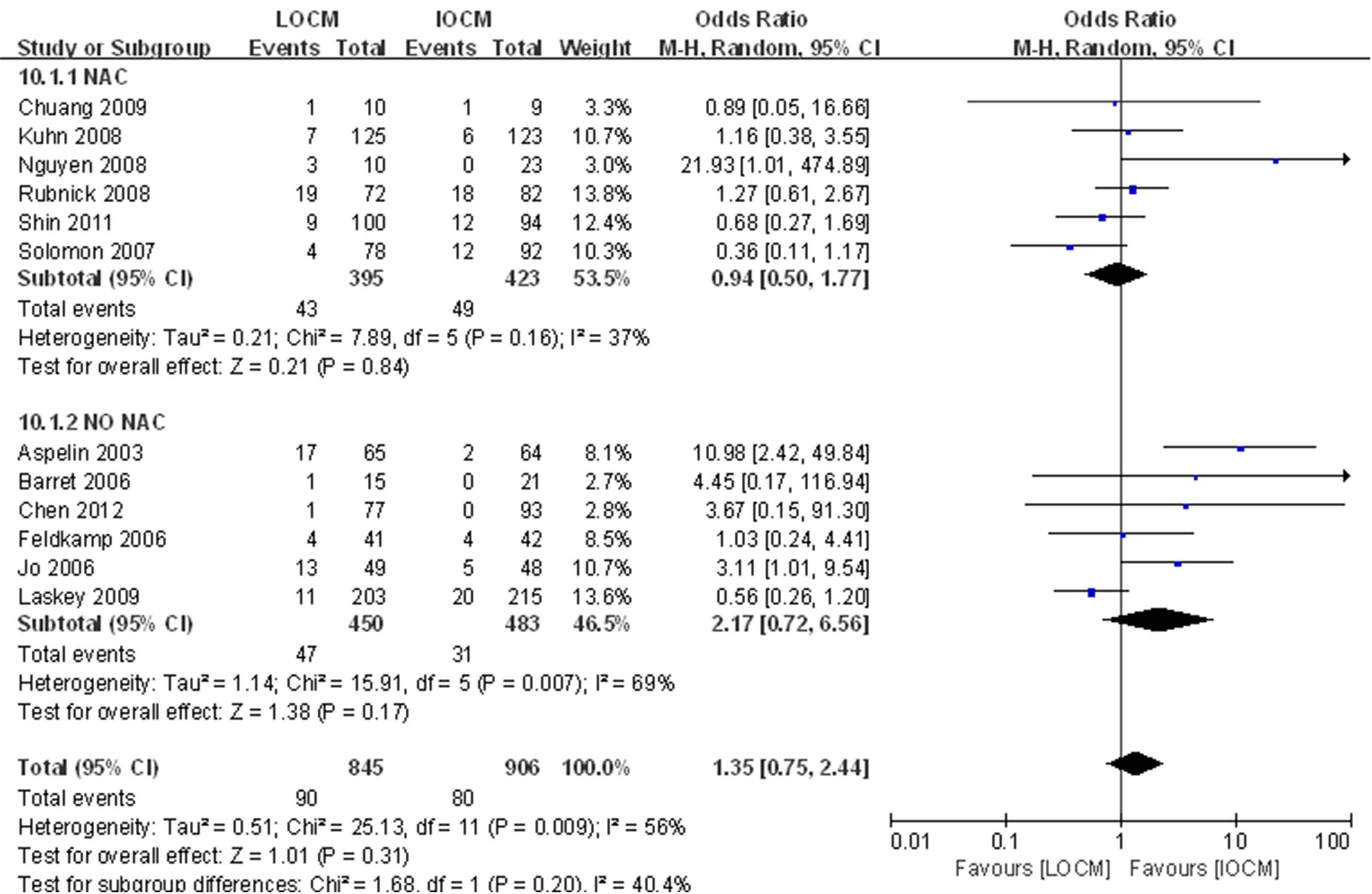

Supplement: Supplementary file 6 — Figure S6. IOCM vs. LOCM for the outcome of CI-AKI: subgroup analysis based on using of NAC. Odds ratio for individual studies (squares) and meta-analysis (diamonds) and 95% CI (horizontal lines) are shown. (TIF 3317 kb) [file 40644_2019_224_MOESM6_ESM.tif]

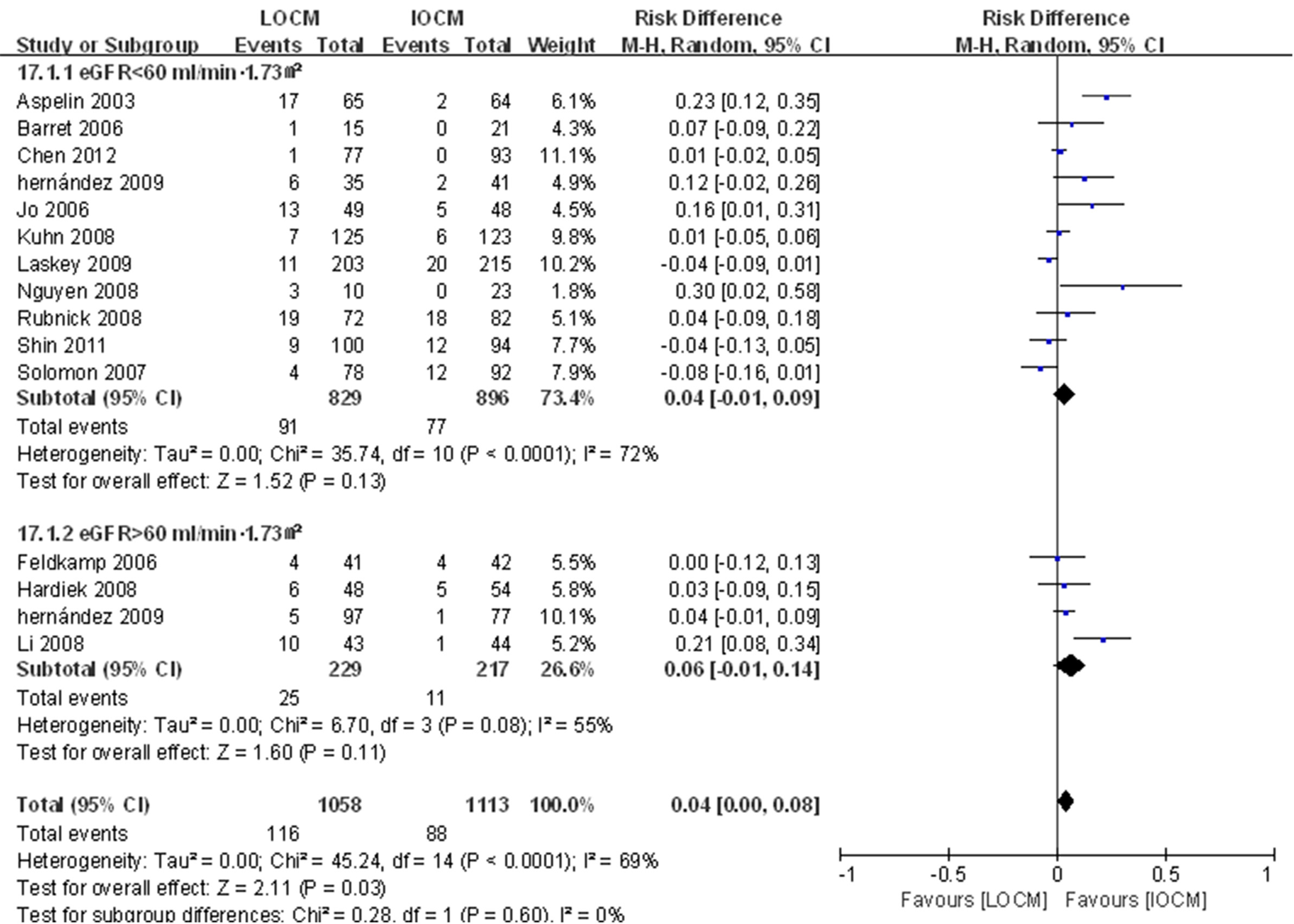

Supplement: Supplementary file 7 — Figure S7. IOCM vs. LOCM for the outcome of CI-AKI: subgroup analysis based on eGFR. Odds ratio for individual studies (squares) and meta-analysis (diamonds) and 95% CI (horizontal lines) are shown. (TIF 3727 kb) [file 40644_2019_224_MOESM7_ESM.tif]

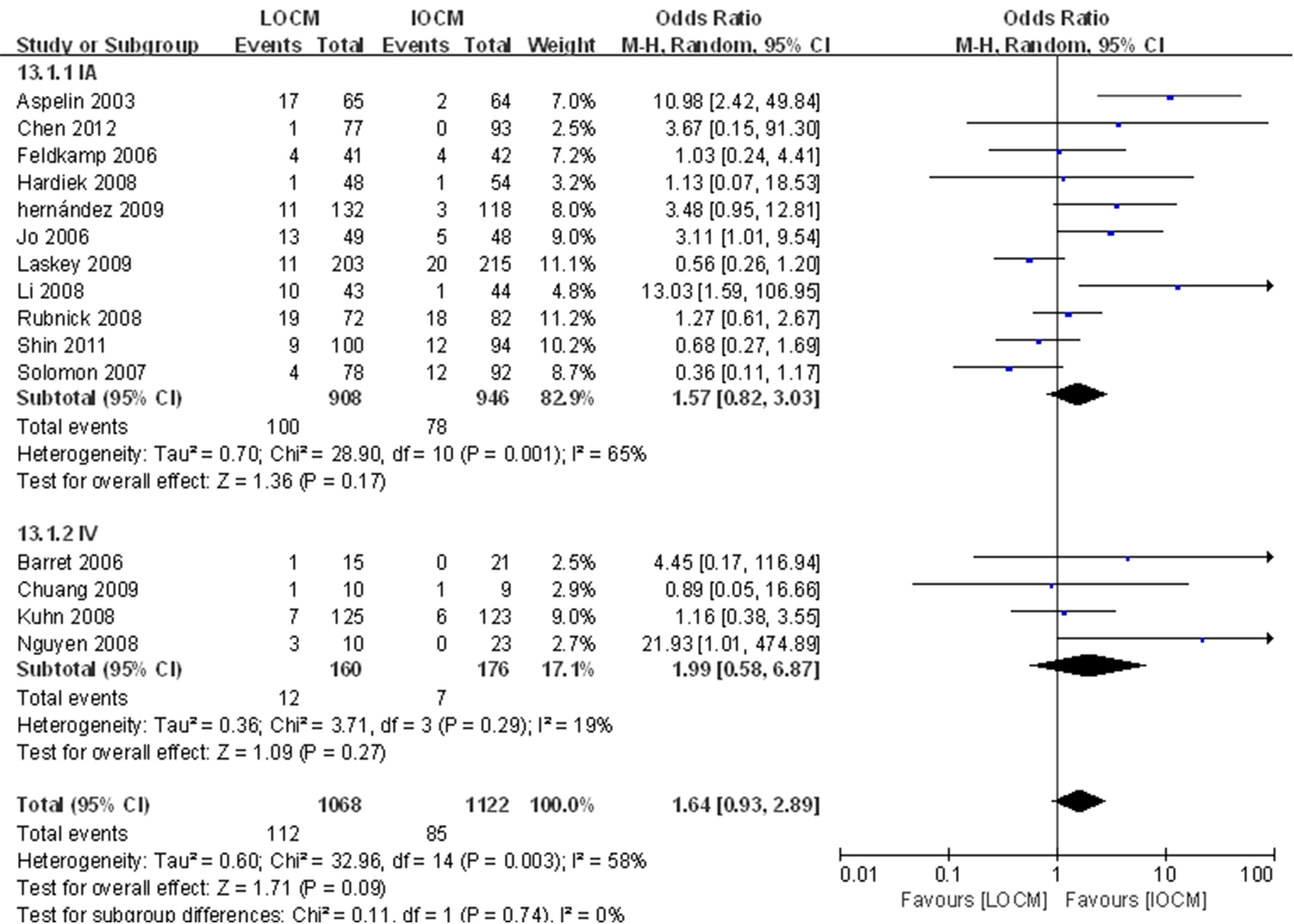

Supplement: Supplementary file 8 — Figure S8. IOCM vs. LOCM for the outcome of CI-AKI: subgroup analysis based on route of administration. Odds ratio for individual studies (squares) and meta-analysis (diamonds) and 95% CI (horizontal lines) are shown. (TIF 3626 kb) [file 40644_2019_224_MOESM8_ESM.tif]

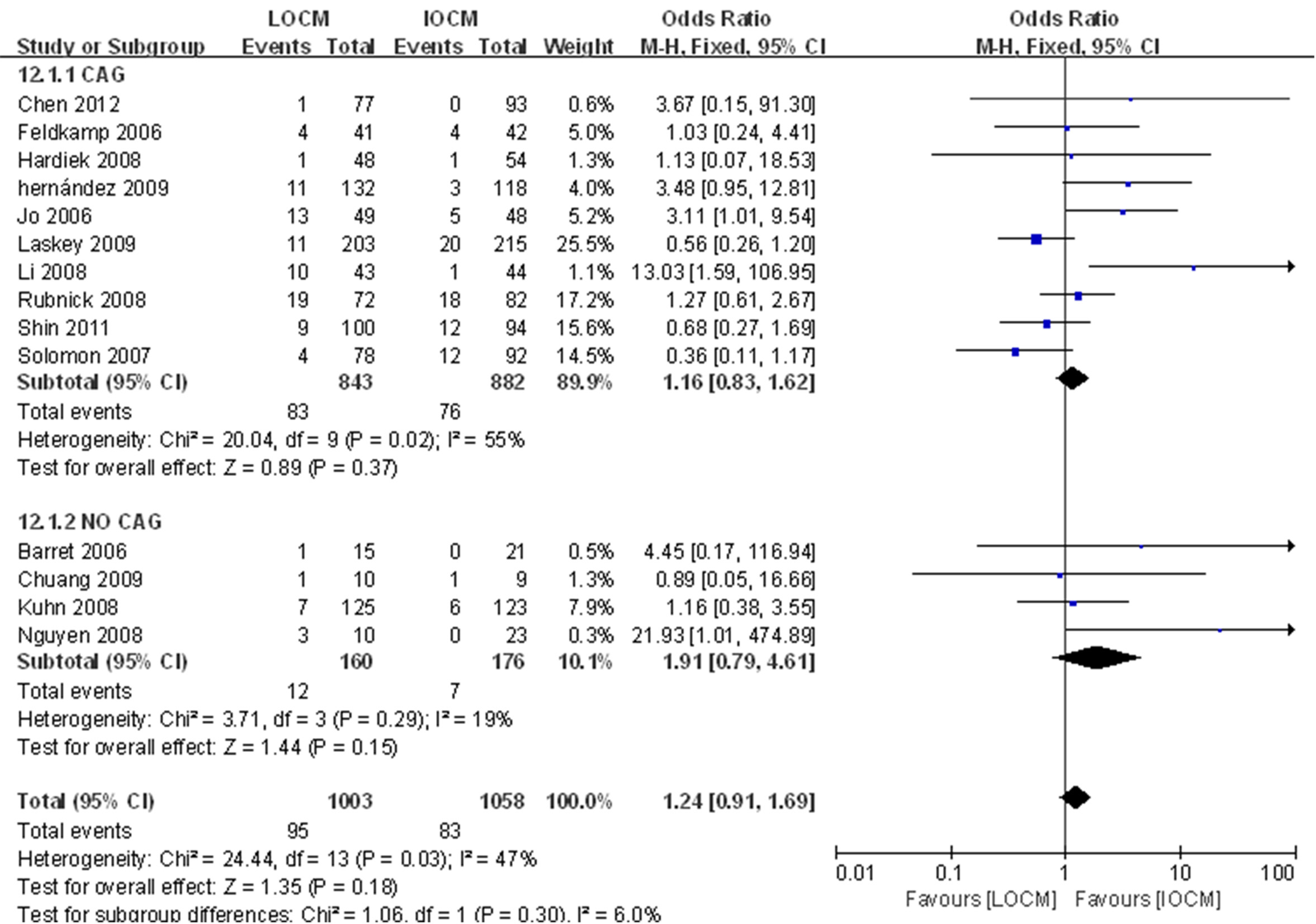

Supplement: Supplementary file 9 — Figure S9. IOCM vs. LOCM for the outcome of CI-AKI: subgroup analysis based on whether or not CAG was performed. Odds ratio for individual studies (squares) and meta-analysis (diamonds) and 95% CI (horizontal lines) are shown. (TIF 3397 kb) [file 40644_2019_224_MOESM9_ESM.tif]

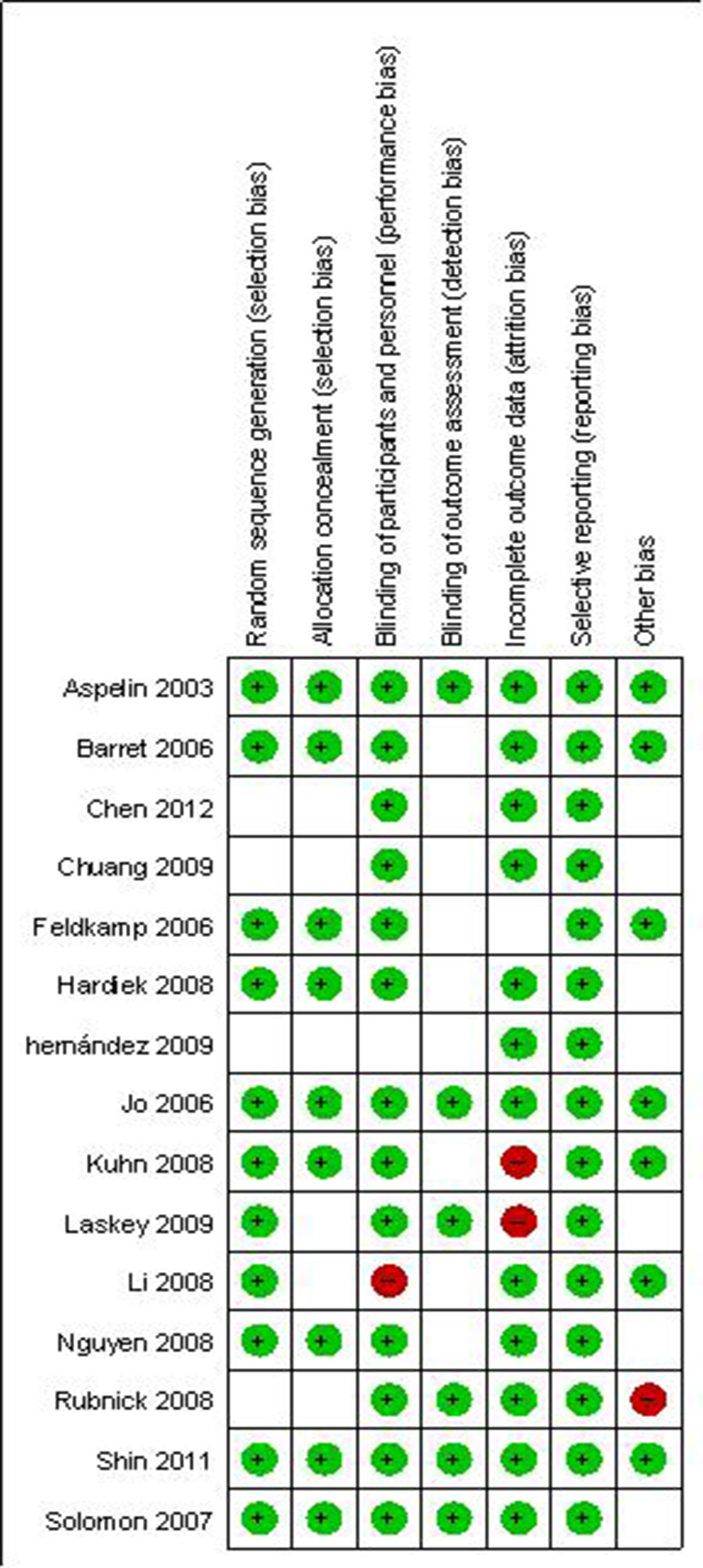

Supplement: Supplementary file 10 — Figure S10. Risk of bias summary: review authors' judgements about each risk of bias item for each included study (TIF 4669 kb) [file 40644_2019_224_MOESM10_ESM.tif]

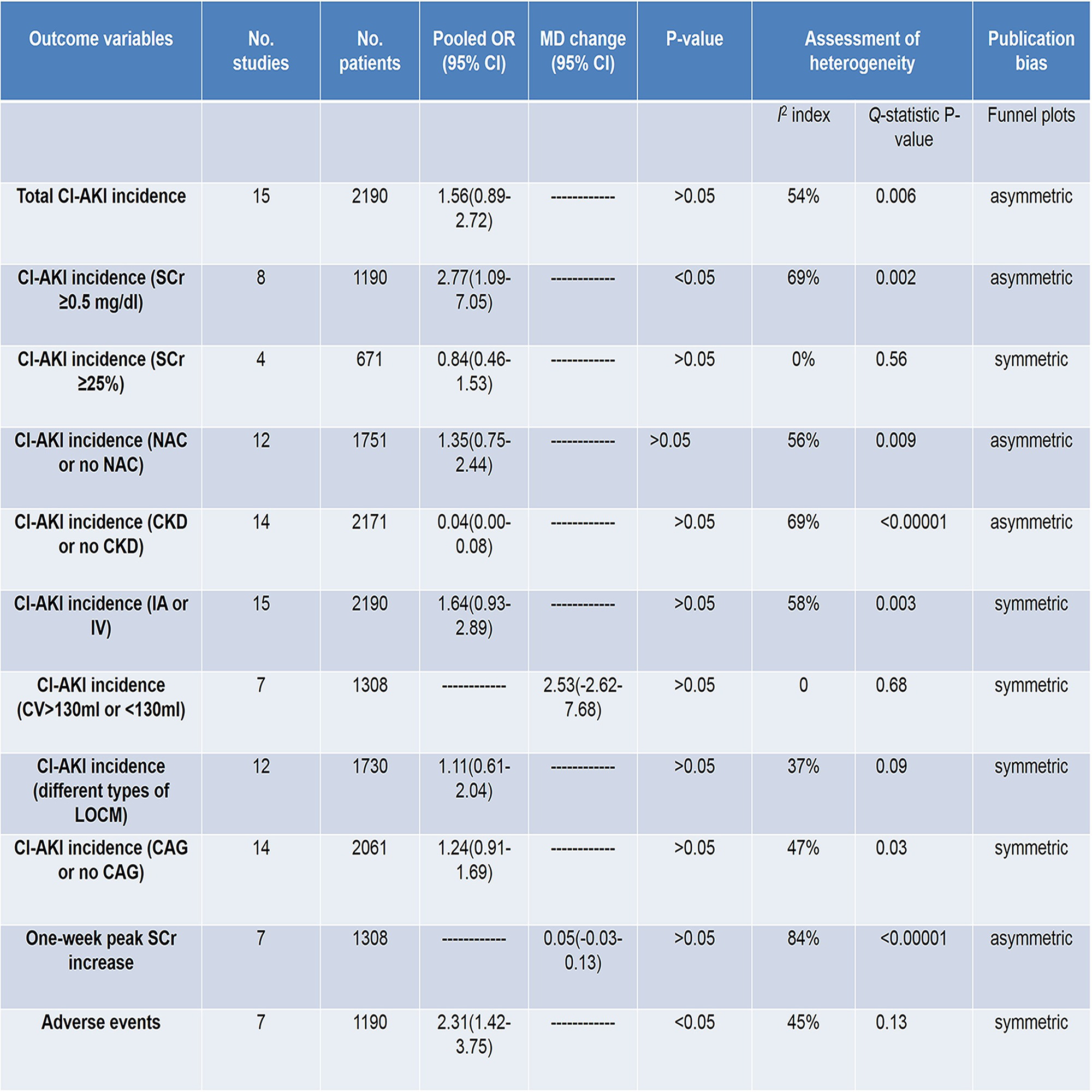

Supplement: Supplementary file 12 — Table S1. Effect of IOCM and LOCM on CI-AKI incidence among diabetic patients. (TIF 2221 kb) [file 40644_2019_224_MOESM12_ESM.tif]
